# The Domain-General Multiple Demand Network Is More Active in Early Balanced Bilinguals Than Monolinguals During Executive Processing

**DOI:** 10.1162/nol_a_00058

**Published:** 2021-12-23

**Authors:** Saima Malik-Moraleda, Theodor Cucu, Benjamin Lipkin, Evelina Fedorenko

**Affiliations:** Department of Brain and Cognitive Sciences, Massachusetts Institute of Technology, Cambridge, MA, USA; McGovern Institute for Brain Research, Massachusetts Institute of Technology, Cambridge, MA, USA; Program in Speech and Hearing Bioscience and Technology, Harvard University, Boston, MA, USA

**Keywords:** bilingualism, bilingual advantage, executive functioning, multiple demand system

## Abstract

The bilingual experience may place special cognitive demands on speakers and has been argued to lead to improvements in domain-general executive abilities, like cognitive control and working memory. Such improvements have been argued for based on both behavioral and brain imaging evidence. However, the empirical landscape is complex and ridden with controversy. Here we attempt to shed light on this question through an fMRI investigation of relatively large, relatively homogeneous, and carefully matched samples of early balanced bilinguals (*n* = 55) and monolinguals (*n* = 54), using robust, previously validated individual-level markers of neural activity in the domain-general multiple demand (MD) network, which supports executive functions. We find that the bilinguals, compared to the monolinguals, show significantly stronger neural responses to an executive (spatial working memory) task, and a larger difference between a harder and an easier condition of the task, across the MD network. These stronger neural responses are accompanied by better behavioral performance on the working memory task. We further show that the bilingual-vs.-monolingual difference in neural responses is not ubiquitous across the brain as no group difference in magnitude is observed in primary visual areas, which also respond to the task. Although the neural group difference in the MD network appears robust, it remains difficult to causally link it to bilingual experience specifically.

## INTRODUCTION

Bilingualism is a growing phenomenon across the globe (e.g., Bacon-Shone & Bolton, 1998; Hoffmann, 2000; Ryan, 2013). How does the ability to speak and understand two or more languages affect our cognitive and neural architecture? Bilingualism necessarily affects linguistic knowledge representations, where instead of a 1:1 mapping between linguistic forms and meanings, a 2:1 mapping exists. Bilingualism also affects the retrieval of linguistic representations in the course of both comprehension and production, given that words and constructions in multiple languages may get activated (e.g., Kaushanskaya & Marian, 2007; Kroll et al., 2014; Thierry & Wu, 2007). More controversially, bilingualism has been argued to affect cognitive abilities beyond language. The claim that has received the most attention in the literature concerns executive abilities. The general reasoning is that switching between languages requires domain-general cognitive control—a core executive ability—and, over time, strengthens this ability (e.g., Abutalebi & Green, 2007; Bialystok, 2017; Declerck & Philipp, 2015; Kroll et al., 2015).

A number of behavioral and brain imaging studies have claimed to provide evidence in support of this *bilingual advantage* in executive functions (e.g., Bialystok, 1999; Bialystok et al., 2005; Grundy & Timmer, 2017; Kapa & Colombo, 2013), and some have even argued that this advantage may have protective benefits in aging and neurodegenerative disorders (e.g., Abutalebi & Green, 2016; Alladi et al., 2013; Antoniou & Wright, 2017; Craik et al., 2010; Guzmán-Vélez et al., 2016; Kroll & Dussias, 2017). However, a growing number of investigations have now challenged these claims, failing to observe a behavioral advantage on executive function tasks (e.g., Antón et al., 2014; Duñabeitia et al., 2014; Lehtonen et al., 2018; Paap & Greenberg, 2013), including in massive samples of thousands of participants (Nichols et al., 2020). Without a robust behavioral manifestation, neural differences between bilinguals and monolinguals may be difficult to interpret. But whether such neural differences exist is also not yet clear.

Although a number of studies have reported differences in activation between bilinguals and monolinguals, different studies have used different paradigms and have reported effects in diverse brain regions (see Luk et al., 2012, for a meta-analysis, and Tao et al., 2021, for a review). In particular, neural differences have been reported in the left and right inferior and middle frontal gyri (e.g., Gold et al., 2013; Mohades et al., 2014; Rodríguez-Pujadas et al., 2013; Teubner-Rhodes et al., 2019), left and right anterior cingulate cortex (e.g., Abutalebi et al., 2013; Gold et al., 2013; Mohades et al., 2014; Waldie et al., 2009), left posterior cingulate cortex (e.g., Mohades et al., 2014), left superior temporal gyrus (e.g., Mohades et al., 2014), and left and right caudate (e.g., Abutalebi et al., 2013; Mohades et al., 2014). Further, in studies where similar brain structures have been implicated, the direction of the effect sometimes differs: For example, Abutalebi et al. (2012) reported lower activations in bilinguals in the anterior cingulate cortex and interpreted this effect as more efficient recruitment, but Mohades et al. (2014) reported stronger activation in bilinguals. More generally, to the best of our knowledge, no direct replications of any reported effect have been carried out (even within the same research group), and publication bias may be “hiding” investigations that have failed to observe a difference (e.g., de Bruin et al., 2015).

Why have we not arrived at a clear and consistent answer about whether bilinguals have superior executive function abilities? One general source of complexity that likely affects both behavioral and brain imaging studies has to do with the nature of the population in question. Bilingualism is a heterogeneous phenomenon (e.g., Luk & Bialystok, 2013; Zirnstein et al., 2019): Bilinguals differ in how early and by what means they acquire their languages, the relative proficiencies and proportions of daily use for each language, and whether they live in a primarily monolingual vs. bilingual environment. The latter factor, in particular, was recently hypothesized to importantly affect executive functions in bilinguals: Perhaps only bilinguals living in primarily monolingual environments and thus having to switch between languages based on environmental constraints would exhibit a bilingual executive advantage (Blanco-Elorrieta & Pylkkänen, 2018). Efforts are ongoing to better characterize the variability in the bilingual population and to relate this variability to brain structure and function (e.g., de Bruin, 2019; Del Maschio & Abutalebi, 2019; Deluca et al., 2019; Gallo et al., 2021; Sulpizio et al., 2020; Zirnstein et al., 2019). Whether or not differences among the samples of bilingual populations used in prior studies can explain the inconsistencies of observing vs. not observing a bilingual executive advantage remains to be determined (García-Pentón et al., 2016).

In terms of prior neural studies reporting a bilingual executive advantage, a number of methodological limitations have plausibly contributed to the complex empirical landscape that has emerged, and to the difficulty of interpreting and evaluating the robustness of the reported effects. Before highlighting some of these issues, let us consider what would constitute neural evidence for a bilingual executive advantage. *Where* would we expect to find the effect? Given the nature of the claim, we would expect to observe a difference between bilinguals and monolinguals in a brain region or regions that have been linked to executive functions. The prime candidate is the bilateral frontoparietal domain-general multiple demand (MD) network (Assem, Blank, et al., 2020; Duncan, 2010, 2013; Duncan et al., 2020). Activity in this network has been reported for diverse demanding cognitive tasks, with stronger responses to more demanding conditions (e.g., Duncan & Owen, 2000; Fedorenko et al., 2013; Hugdahl et al., 2015; Shashidhara et al., 2020) and linked to cognitive constructs like attention, working memory, cognitive control, and fluid intelligence. In the behavioral literature, different aspects of executive abilities have been argued to be at least partially dissociable (e.g., Miyake et al., 2000). However, how these alleged dissociations may be implemented in the brain remains debated. Given strong interregional correlations in neural activity among the MD regions (e.g., Assem, Blank, et al., 2020; Assem, Glasser, et al., 2020; Blank et al., 2014; Braga et al., 2020; Mineroff et al., 2018; Paunov et al., 2019; Power et al., 2011; Yeo et al., 2011), we here consider the MD network to be a functionally integrated system and executive functions to be a host of interrelated abilities.

What about the *direction* of the effect? Should we expect the MD network to be more active or less active in individuals with superior executive abilities? Prior work has compellingly established that stronger MD responses are associated with better behavioral performance both within and across individuals (e.g., Assem, Glasser, et al., 2020; Basten et al., 2013; Burgess et al., 2011; Choi et al., 2008; Cole et al., 2012; Gray et al., 2003; Lee et al., 2006; Tschentscher & Mitchell, 2017). So, if bilinguals were better at (some aspect of) executive functions, we would expect to observe stronger activation—relative to a matched group of monolinguals—within the domain-general MD network for a task targeting executive functions. This neural difference should further be accompanied by better performance in the form of higher accuracies and/or faster reaction times.

To motivate the current study, let us now highlight several issues that have plagued prior brain imaging studies of executive functions in bilinguals (for reviews, see Costa & Sebastián-Gallés, 2014; Pliatsikas & Luk, 2016; Tao et al., 2021). First, most past studies have relied on “reverse inference” reasoning (Fedorenko, 2021; Poldrack, 2006, 2011)—from anatomy to function—to interpret the observed effects. For example, many studies have reported effects somewhere in the left frontal cortex (e.g., Gold et al., 2013; Mohades et al., 2014; Rodríguez-Pujadas et al., 2013; see Luk et al., 2012 for a meta-analysis) and argued that these effects reflect differences in executive functions given that many executive function tasks activate frontal areas. However, this reasoning is not valid: Left frontal cortex is structurally and functionally heterogeneous and contains subsets of at least two distinct brain networks (e.g., Fedorenko et al., 2012; see Fedorenko & Blank, 2020, for a review). One of these is the network of interest—the MD network, but the other is the language-selective network (e.g., Braga et al., 2020; Fedorenko et al., 2011; Fedorenko & Thompson-Schill, 2014), which does not support executive functions. Given the well-documented interindividual variability in the precise locations of the MD and language areas (e.g., Fedorenko et al., 2011, 2013; Shashidhara et al., 2020), an anatomical location cannot be used to interpret an effect as arising within the MD network vs. the language network.

Second, to the best of our knowledge, all prior work has relied on comparisons of group-level activation maps. In such analyses, individual maps in each group are aligned in the common brain space, and voxel-wise functional correspondence is assumed to hold across participants, and the group-level maps for bilinguals and monolinguals are then compared. Such analyses suffer from limited sensitivity and functional resolution (Nieto-Castañón & Fedorenko, 2012) due to interindividual differences in the precise locations of the functional regions (see Shashidhara et al., 2020, for evidence of such variability for the MD network in particular). In cases of between-group comparisons, this variability can lead to misleading, and even altogether opposite, patterns of results. For example, imagine that the functional topography is less variable in the monolingual population, leading to better alignment at the group level. In this scenario, even if at the individual level, every bilingual individual shows stronger effects than every monolingual individual, the group-level comparison will show a more pronounced effect in the monolingual group, which is the opposite of the true effect.

Third, most prior neuroimaging studies of bilinguals have relied on small, and sometimes heterogeneous, samples, which can lead to spurious effects driven by a small number of outliers (e.g., Assem, Glasser, et al., 2020).

Finally, in order to ensure that an observed effect in the MD network is not due to a group-level difference in variables that would affect responses *across the brain*, such as brain vascularization (e.g., Erdogan et al., 2016; He et al., 2010; Poldrack, 2011), motion (e.g., Hajnal et al., 1994; Power et al., 2015), vigilance levels (e.g., Wong et al., 2013), or arousal (e.g., Chang et al., 2016; Schölvinck et al., 2010), it is important to demonstrate that any group difference observed between bilinguals and monolinguals in the MD system is not present in some control brain region, as supported by a region by group interaction (e.g., Nieuwenhuis et al., 2011). To the best of our knowledge, none of the past studies have included such control regions.

In an effort to bring clarity to the ongoing debate about whether or not bilingual individuals have superior executive abilities, we carried out an fMRI investigation where we (i) localized the network of interest (the MD network) in each individual participant using a well-established paradigm (a spatial working memory task) that has been previously shown to activate the same areas as other diverse executive-function tasks (e.g., Fedorenko et al., 2013; Shashidhara et al., 2020) and to robustly isolate the MD network from the language network (Blank et al., 2014; Fedorenko et al., 2012, 2013; Ivanova et al., 2020; Mineroff et al., 2018); (ii) examined individual-level neural markers (magnitudes of response to the target task, estimated using data independent from the data used to localize the regions of interest) that have been shown to be stable within individuals over time and to correlate with behavioral performance (Assem, Glasser, et al., 2020); (iii) included a control set of regions—primary visual areas—to evaluate the spatial specificity of the effect; and (iv) examined a relatively large (*n* = 55) and relatively homogeneous set of bilinguals (early balanced bilinguals who live in an English-speaking country—the United States; see Figure 2a for details), matched carefully to a similarly sized group of monolinguals (see Table 1 for details).

**Table 1.** Summary of the variables for which the two groups were matched.

**Group**	**Age mean (*SD*)**	**% Female**	**% Right-handed**
Bilingual	25.47 (4.87)	43.6%	81.8%
Monolingual	25.42 (5.81)	48.1%	87.0%

## MATERIALS AND METHODS

### Participants

The study included 109 participants: 55 bilinguals and 54 monolinguals. Participant selection proceeded as follows. First, 87 bilingual–monolingual pairs of participants were identified among the 800+ participants in the Fedorenko Lab’s database, the majority of whom had completed the task of interest (the spatial working memory task). These pairs were selected so as to be similar in age and have the same gender and handedness. Next, 11 participants were removed (6 bilingual, 5 monolingual) because they had completed only one run of the task (two runs are necessary to estimate the response magnitudes in individually defined functional regions of interest (fROIs); see below for details); and 14 additional participants were removed (1 bilingual, 13 monolingual) due to data quality issues. These exclusions left 149 participants (80 bilingual and 69 monolingual). Finally, following feedback from the reviewers, 40 additional participants were removed (25 bilingual, 15 monolingual) in order to ensure that (i) all bilingual participants learned their second language before the age of 6 and reported a proficiency score of 4 or 5 on a scale from 1 to 5 (see below for details), and that (ii) all monolingual participants that reported having studied any foreign language in school did so after the age of 10 and reported a proficiency score of 1 or 2. (See Supporting Information 1, which can be found at https://doi.org/10.1162/nol_a_00058.) These exclusions left 109 participants (55 bilingual, 54 monolingual). In the final set, 32 of the original 87 pairs remained, with the other 45 participants not being pairwise matched. However, the two groups remained well-matched on age (*p* = 0.86), gender (*p* = 0.92), and handedness (*p* = 0.98; see Table 1).

Participants in the bilingual group were native speakers of diverse languages (see Table SI-1 for detailed language profiles of all participants) and reported speaking two (*n* = 17), three (*n* = 24), or four or more (*n* = 14) languages. Crucially, as noted above, all participants acquired their second language at an early age (mean = 2.14 years, *SE* = 0.30), and on a scale from 1 (no knowledge) to 5 (native-like proficiency), they self-reported speaking their second language with high proficiency (mean = 4.91, *SE* = 0.03; Figure 2a). The majority (*n* = 44) listed English as their second language or as one of two languages acquired simultaneously from birth, while the rest (*n* = 11) listed a different language as their second language (Table SI-1) and English as their third language. Participants in the monolingual group were native English speakers; the majority did not report having studied a second language (*n* = 35), and the rest (*n* = 19) reported learning a second language at school and relatively late in life (mean = 13.84 years, *SE* = 0.62) and self-reported a low proficiency level (mean = 1.8, *SE* = 0.04) (Figure 2a and Table SI-1).

Participants had normal or corrected-to-normal vision. All participants gave informed consent as required by the Committee on the Use of Humans as Experimental Subjects (COUHES; https://couhes.mit.edu/) and were paid for their participation.

### Experimental Design

**Figure 1.**
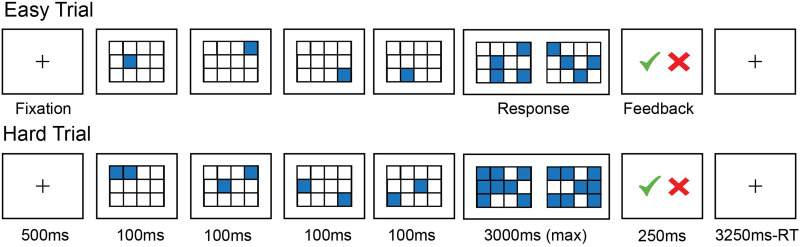
Sample trials of the Easy and Hard conditions of the spatial working memory task.

Every participant completed a spatial working memory task as part of a 2-hr fMRI scanning session for one of the projects in the Fedorenko Lab. This task is routinely used in the lab as a localizer for the domain-general MD system (Assem, Blank, et al., 2020; Duncan, 2010, 2013; Duncan et al., 2020; Fedorenko et al., 2013). In this task, participants are presented with a 3 × 4 grid, and on each trial, they see a sequence of locations flash up within the grid. In the Easy condition, locations appear one at a time for a total of four locations, and in the Hard condition, locations appear two at a time for a total of eight locations. After the sequence, participants are presented with two grids showing two different sets of locations and have to indicate which set of locations they had just seen. The grid with the incorrect set of locations has one or two incorrect locations. Participants are given feedback on whether they chose correctly in the form of a green checkmark or a red “X.” Each trial lasts 8 s (see Figure 1 for details of the timing), and trials are grouped into blocks of four. Each run consists of twelve 32-s-long experimental blocks (six per condition) and four 16-s-long fixation blocks for a total run duration of 448 s (7 min 28 s). All participants completed two runs (for a total task duration of ∼15 min), with condition order counterbalanced across runs.

### fMRI Data Acquisition

A whole-body 3 Tesla Siemens Trio scanner with a 32-channel head coil was used to collect both structural and functional data. The structural images were collected with 1 mm isotropic voxels (TR = 2,530 ms, TE = 3.48 ms) in 179 sagittal slices. An echo-planar imaging sequence (flip angle: 90°, GRAPPA with 2 times acceleration factor) was used for the acquisition of functional BOLD signal. The acquisition parameters were as follows: 31 4-mm thick near-axial slices, in an interleaved order with a 10% distance factor; 2.1 mm × 2.1 mm in-plane resolution; field of view of 200 mm in the phase encoding anterior to posterior (A > P) direction; matrix size of 96 × 96; TR of 2,000 ms; and TE of 30 ms. The gradient positioning based on participant’s motion was adjusted using prospective acquisition correction. In order to allow for the magnetization to become steady state, the first 10 s of each run were discarded.

### fMRI Data Preprocessing and First-Level Analysis

fMRI data were analyzed using SPM12 (Wellcome Centre for Human Neuroimaging, UCL Queen Square Institute of Neurology, London, UK; https://www.fil.ion.ucl.ac.uk/spm/software/spm12/) and custom MATLAB (https://www.mathworks.com/) scripts. SPM was used for preprocessing and first-level data modeling. Each participant’s data were motion corrected and then normalized into a common brain space (the Montreal Neurological Institute (MNI) template) and resampled into 2-mm isotropic voxels. The data were then smoothed with a 4-mm Gaussian filter and high-pass filtered (at 128 s). To model the spatial working memory task, a standard mass univariate analysis was performed whereby a general linear model estimated the effect size of each condition in each experimental run. These effects were each modeled with a boxcar function (representing entire blocks) convolved with the canonical hemodynamic response function. The model also included first-order temporal derivatives of these effects, as well as nuisance regressors representing entire experimental runs, offline-estimated motion parameters, and time points classified as outliers during the preprocessing (i.e., scans where the scan-to-scan differences in global BOLD signal are above 5 standard deviations, or where the scan-to-scan motion is above 0.9 mm).

### MD fROI Definition and Response Estimation

For each participant, fROIs were defined using the *Group-constrained subject-specific (GSS) approach* (Fedorenko et al., 2010). In this approach, a set of *masks* (or parcels) delineating brain areas, within which most individuals in prior studies had shown activity for the localizer contrast, are combined with each individual participant’s activation map for the same contrast. Here, for comparability with Fedorenko et al. (2013) and Assem, Glasser, et al. (2020), we used a set of eighteen anatomical parcels; these covered the bilateral frontal and parietal brain regions that have long been implicated in executive functions (e.g., Cabeza & Nyberg, 2000; Duncan, 2010, 2013) and included the middle frontal gyrus (MFG; 4,863 voxels LH, 5,104 voxels RH), the orbital part of the middle frontal gyrus (MFGorb; 888 voxels LH, 1,015 voxels RH), the inferior frontal gyrus (IFG; 1,038 voxels LH, 1,399 voxels RH), the precentral gyrus (PrecG; 3,528 voxels LH, 3,381 voxels RH), the supplementary motor area (SMA; 2,147 voxels LH, 2,371 voxels RH), the anterior cingulate cortex (ACC; 1,615 voxels LH, 1,958 voxels RH), the superior parietal cortex (ParSup; 2,065 voxels LH, 2,222 voxels RH), the inferior parietal cortex (ParInf; 2,447 voxels LH, 1,345 voxels RH), and the insula (1,858 voxels LH, 1,770 voxels RH). The masks are available for download from: evlab.mit.edu/funcloc/.

For each individual participant, MD fROIs were defined by selecting 10% of voxels within each parcel that were most responsive to the *Hard* > *Easy* spatial working memory contrast, as defined by their *t* values. To estimate the responses of these fROIs to the Easy and Hard conditions, an across-runs cross-validation procedure was used (Nieto-Castañón & Fedorenko, 2012): first, run 1 of the localizer was used to define the fROIs, and run 2 to estimate the responses (in percent BOLD signal change) to the localizer condition, ensuring independence (Kriegeskorte et al., 2009); second, run 2 was used to define the fROIs, and run 1 to estimate the responses; finally, the extracted magnitudes were averaged across the two runs to derive a single response magnitude per condition (hard and easy spatial working memory) per fROI per participant.

### Control Regions

To test whether the group difference in response to the spatial working memory task that may be observed within the MD network is present across the brain, we selected a set of control brain regions. In particular, we used three bilateral anatomical parcels (from Tzourio-Mazoyer et al., 2002) that cover primary visual areas. The rationale for selecting visual areas was that they should show strong responses to the spatial working memory task given its visual nature, so the comparison with the MD fROIs was fair. For this analysis, the responses to the Easy and Hard conditions of the task were estimated across all voxels in each parcel in each participant, and then averaged across the voxels in each parcel to obtain a single estimate per condition per parcel per participant.

### Statistical Analyses

The data were analyzed with linear mixed-effect models using the lme4 package in R (https://cran.r-project.org/web/packages/lme4/index.html); *p*-value approximation was performed with the lmerTest package, while effect sizes were calculated with the rstatix package (Bates et al., 2015; Kuznetsova et al., 2017). The following linear mixed-effect regression models were fit in order to address three critical research questions (all the analysis scripts and the data tables are available at OSF [https://osf.io/b6xjy/]):

(a)  Does the MD network respond differentially in bilinguals and monolinguals during an executive (spatial working memory) task?

The BOLD response was predicted by a model that included two fixed effects: condition (*Hard* (relative to fixation), *Easy* (relative to fixation), and *Hard* > *Easy*) and group (bilingual and monolingual). ROIs (*n* = 18) and participants (*n* = 109) were modeled as random effects with random intercepts. ROIs were included as a random effect instead of a fixed effect because, as discussed in the Introduction, the regions in the MD network have been previously reported to be strongly functionally integrated, as evidenced by a high degree of synchronization during naturalistic cognition (Assem, Blank, et al., 2020; Blank et al., 2014; Braga et al., 2020; Paunov et al., 2019) and strong interregional correlations in effect sizes (Assem, Glasser, et al., 2020; Mineroff et al., 2018). However, for completeness, in Supporting Information 2, we report models estimated for each ROI separately.

EffectSize∼Condition+Group+Group*Condition+(1|ROI)+(1|Participant)

(b)  Do bilinguals perform better than monolinguals behaviorally on the spatial working memory task?

The accuracy and reaction times on the spatial working memory task were predicted by two separate models that included a fixed effect for group (bilingual and monolingual). Participants (*n* = 65; 28 bilingual, 37 monolingual) were included as random effects with random intercepts. (Note that the behavioral data for the remaining 44 participants (27 bilingual, 17 monolingual) were not collected due to experimenter error or equipment malfunction, or were lost/overwritten.)

$$\text{Accuracy}\left(\text{or}\;RT\right)∼\text{Group}+(1|\text{Participant})$$

(c)  Do the control (primary visual) areas respond differentially in bilinguals and monolinguals during the spatial working memory task, and do the MD network and the primary visual areas differ in their responses?

First, the BOLD response was predicted by a model that included two fixed effects: condition (*Hard* (relative to fixation), *Easy* (relative to fixation), and *Hard* > *Easy*) and group (bilingual and monolingual). ROIs (*n* = 18) and participants (*n* = 109) were modeled as random effects with random intercepts.

EffectSize∼Condition+Group+Group*Condition+(1|ROI)+(1|Participant)

Next, to explicitly test whether the MD network and the primary visual areas differ in their responses between the two groups (e.g., Nieuwenhuis et al., 2011), the BOLD response was predicted by a model that included four fixed effects: condition (*Hard* (relative to fixation), *Easy* (relative to fixation), and *Hard* > *Easy*), group (bilingual and monolingual), network (MD and Visual), and critically, a group by network interaction. ROIs (*n* = 18) and participants (*n* = 109) were modeled as random effects with random intercepts.

EffectSize∼Condition+Group+Network+Group*Network+(1|ROI)+(1|Participant)

## RESULTS

As expected, and in line with previous research (Assem, Glasser, et al., 2020; Fedorenko et al., 2013), the MD network showed a highly robust Hard > Easy effect across participants (*b* = 0.89, *SE* = 0.19; *p* < 0.001), and in each group separately (bilinguals: *b* = 0.96, *SE* = 0.19; *p* < 0.001; monolinguals: *b* = 0.82, *SE* = 0.19; *p* < 0.001). The critical results were as follows.

(1)  ***The MD network responded more strongly in bilinguals than in monolinguals during an executive (spatial working memory) task.***

**Figure 2.**
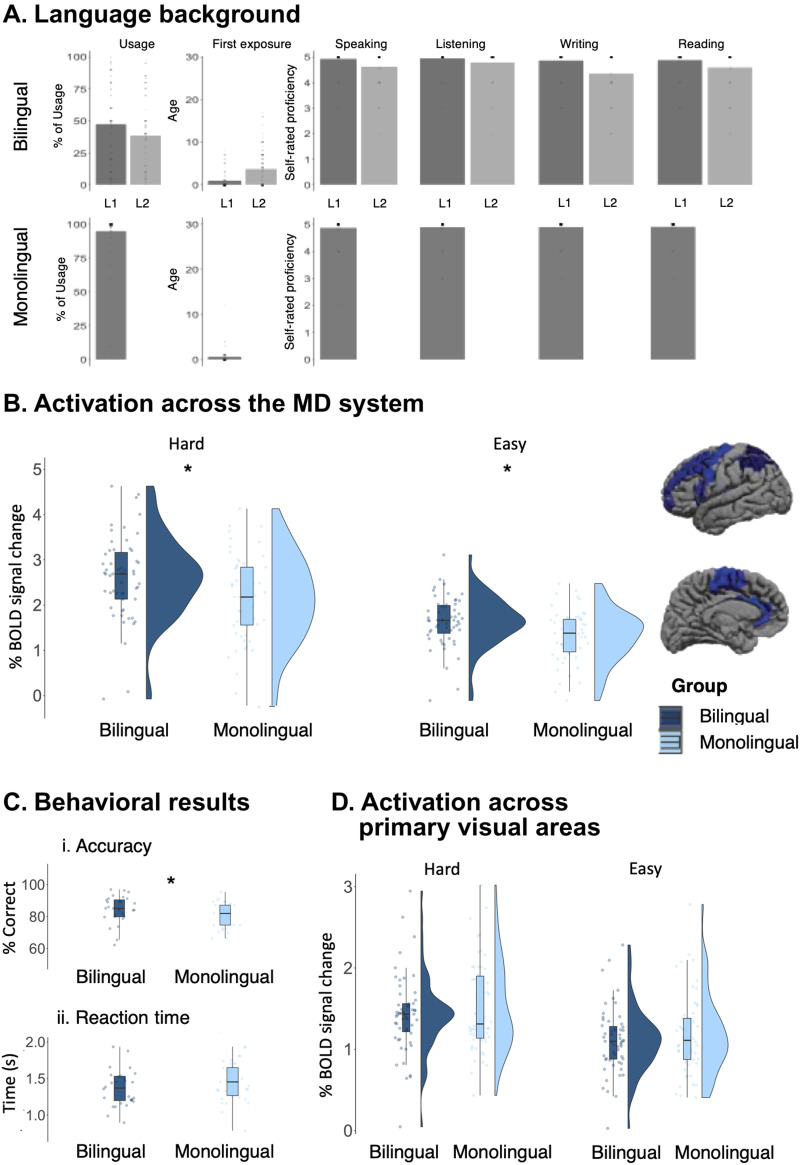
(A) The language background of bilingual and monolingual participants: usage of language (in %), age of first exposure, and self-rated proficiency scores from 1 (no knowledge) to 5 (native-like proficiency) for speaking, listening, writing, and reading are reported. (B) Activation (in % BOLD signal change) across the MD system during the Hard and Easy conditions of the spatial working memory task. (C) Accuracy and reaction times for the spatial working memory task. (D) Activation (in % BOLD signal change) across the primary visual areas during the Hard and Easy conditions of the spatial working memory task.

A significant effect of group was observed: The MD fROIs responded more strongly in the bilingual compared to the monolingual participants during both the Hard condition (bilingual: mean = 2.62, *SE* = 0.02; monolingual: mean = 2.16, *SE* = 0.05; *p* < 0.01; Figure 2b) and the Easy condition (bilingual: mean = 1.66, *SE* = 0.04; monolingual: mean = 1.34, *SE* = 0.04; *p* < 0.01). Further, the Hard > Easy effect was larger in the bilinguals (mean = 0.96, *SE* = 0.02) than in the monolinguals (mean = 0.82, *SE* = 0.02; *p* < 0.001).

(2)  ***Bilinguals performed better than monolinguals behaviorally on the spatial working memory task.***

The bilinguals’ accuracies were higher (mean = 84.8%, *SE* = 1.64) than the monolinguals’ (mean = 79.3%, *SE* = 1.94; *p* = 0.03). Moreover, bilingual participants were numerically faster (mean = 1.33 s, *SE* = 0.04) than monolingual participants (mean = 1.42 s, *SE* = 0.04; *p* = 0.16). Both effects were small (Cohen’s *d* = 0.48 and −0.31, respectively), so we may not have had sufficient power to detect an effect in the reaction time data.

(3)  ***The primary visual areas responded similarly in bilinguals and monolinguals during the spatial working memory task, and the MD network and the primary visual areas differed in their responses.***

Similar to the MD network, the primary visual areas showed a robust Hard > Easy effect across participants (*b* = 0.33, SE = 0.08; *p* < 0.001), and in each group separately (bilinguals: *b* = 0.32, *SE* = 0.09; *p* < 0.01; monolinguals: *b* = 0.33, *SE* = 0.09; *p* < 0.01). This is to be expected given that the Hard condition contains more visual information (two squares, compared to one square, for each trial component; see Figure 1). Critically, the primary visual areas of the bilingual participants responded similarly to those of the monolingual participants during the Hard condition (bilingual: mean = 1.41, *SE* = 0.09; monolingual: mean = 1.50, *SE* = 0.11; *p* = 0.78) and the Easy condition (bilingual: mean = 1.10, *SE* = 0.08; monolingual: mean = 1.17, *SE* = 0.10; *p* = 0.35). Further, the size of the Hard > Easy contrast was similar between the groups (bilingual: mean = 0.32, *SE* = 0.03; monolingual: mean = 0.33, *SE* = 0.04; *p* = 0.32). Moreover, a significant group by network interaction obtained (*b* = 0.36, *SE* = 0.04; *p* < 0.001), such that the bilingual vs. monolingual difference in the size of the Hard > Easy effect was reliably larger in the MD network compared to the primary visual areas.

## DISCUSSION

To address the question of whether bilingual individuals exhibit superior executive abilities—an area of investigation characterized by a complex and controversial empirical landscape—we carried out an fMRI investigation that had several unique design features, relative to the past studies, that gave it a stronger chance to obtain a clearer answer, as elaborated in the Introduction and below. We found stronger responses to the spatial working memory task, and a larger difference between a harder and an easier condition, in the bilingual participants compared to the monolingual participants in the MD network, but not in the control (primary visual) areas. This neural difference was accompanied by numerically better behavioral performance. In the remainder of the discussion, we highlight a few implications of these results and some questions they raise, and talk about the limitations of the current investigation.

### The Nature of the Superior Executive Abilities in Bilingual Individuals

We report effects that are reliable and substantial in size such that the bilinguals’ MD network was overall more active during an executive (spatial working memory) task than the MD network in age-, gender-, and handedness-matched monolingual participants, and the difference between the harder and the easier conditions of the task was larger in bilinguals than in monolinguals. What do these effects reflect? Historically, behavioral and neural differences in executive abilities between bilinguals and monolinguals have been attributed to differences in their linguistic experiences: in particular, to the bilinguals’ need to switch between languages, and consequent improvements in their general cognitive control abilities. However, attributing these effects specifically to the differences between the two groups in their linguistic experience is difficult. (This interpretive challenge is also present in all prior studies, even if they have not explicitly acknowledged it.) In particular, bilingual individuals (or at least the type of bilinguals examined here: most individuals are living in a country where their second language is the dominant language, the majority of whom came to the United States to pursue educational and/or professional goals) may be more motivated (Baker, 1988) and/or have higher grit (e.g. Credé et al., 2017). The relationship between such factors and executive abilities remains controversial (e.g., Ebbesen, 2020; Pessoa, 2009; Taylor et al., 2004; Uddin, 2021); whereas some studies control for education and immigration status (Alladi et al., 2013), at present, it is not possible to rule out the potential contribution of such factors to the effects observed here.

General fluid intelligence is also worth a mention: We only had IQ scores on a small subset of our participants, so we could not match the groups for IQ. However, it is actually unclear whether matching on IQ makes sense in evaluating individual differences in executive abilities given the intimate link between fluid intelligence and executive functions (e.g., Assem, Blank, et al., 2020; Duncan, 2010, 2013; Duncan et al., 2020; Gläscher et al., 2010; Woolgar et al., 2010). Indeed, damage to the MD network has been shown to lead to deficits in executive functions as well as to loss of fluid intelligence abilities (see Duncan, 2020, for an extensive discussion), and stronger responses in the MD network have been associated with both better performance on executive tasks and higher IQ scores (e.g., Assem, Glasser, et al., 2020; Basten et al., 2013; Burgess et al., 2011; Choi et al., 2008; Cole et al., 2012; Gray et al., 2003; Lee et al., 2006; Tschentscher & Mitchell, 2017).

These interpretive challenges call for further studies across diverse bilingual populations. If these effects hold across different kinds of bilinguals, that would help rule out potential explanations in terms of motivation/grit, or establish that superior executive abilities characterize only some bilingual/multilingual populations (e.g., Blanco-Elorrieta & Pylkkänen, 2018). For example, it is worth noting that using the same paradigm as the one used here, Jouravlev et al. (2021) found no difference in the neural responses in the MD network in a set of 17 polyglots and hyperpolyglots, most of whom acquired their non-native languages post critical period, as compared to a matched set of monolingual controls. However, in Jouravlev et al.’s study, polyglots and monolinguals were matched for IQ, which as noted above, may not be the right approach when probing for individual differences in executive functions.

To conclusively link superior executive abilities to linguistic experience, longitudinal developmental studies will be critical. In particular, tracking executive abilities in a population of young monolingual children some of whom proceed to acquire a second language (e.g., through a language immersion program) and some of whom do not would be extremely valuable. Of course, longitudinal studies are notoriously challenging, and full experimental control over which subset of children become bilingual may be hard or impossible to achieve.

### Methodological Considerations in Future Studies of Bilingualism

Several unique features of the current study may have enabled us to detect a clear and robust effect, and we hope some of these practices will become more widely adopted in the field of bilingualism research. Perhaps most importantly, we identified the network of interest (the MD network) functionally in each individual participant using a robust MD localizer paradigm. There are three key advantages to this approach. First, functional localization has long been established to vastly improve sensitivity (i.e., the ability to detect an effect; e.g., Brett et al., 2002; Fedorenko et al., 2010; Nieto-Castañón & Fedorenko, 2012; Saxe et al., 2006). This issue is especially pertinent when examining high-level cognitive processes. Such processes are supported by the association cortex, where functional areas (i) are not predictable from macroanatomy (e.g., Frost & Goebel, 2012; Tahmasebi et al., 2012; Vázquez-Rodríguez et al., 2019), and (ii) vary substantially across individuals in their precise locations in a common brain space (e.g., Fedorenko et al., 2010, 2013; Shashidhara et al., 2020). An inevitable consequence is that many effects may be robustly present in each individual participant but would be missed in a standard group analysis, which relies on voxelwise alignment across individuals (note that the use of larger samples does not help with this problem). The use of this low-power analytic approach may explain why prior studies have reported effects in only a subset of the MD network. (Incidentally, arguments that *only* regions where an effect emerged in a traditional group analysis, but not other regions, show the effect of interest are fallacious for the reasons above. In particular, region A but not region B may emerge in a group analysis because region A is better aligned with anatomic landmarks; see, e.g., Blank et al., 2016, for discussion.) The use of this approach may also obscure between-population differences.

Second, functional localization confers a substantial interpretive advantage, removing the need for precarious reverse inference (e.g., Fedorenko, 2021; Poldrack, 2006). In particular, by functionally identifying a network that has been robustly linked to executive functions across diverse tasks (e.g., Fedorenko et al., 2013; Hugdahl et al., 2015; Shashidhara et al., 2020) the observed effects can be straightforwardly interpreted as reflecting differences in *executive functions*. Because the cortex is highly functionally heterogeneous, and distinct areas often lay adjacent to one another within the same macroanatomic area, interpreting effects functionally based on coarse macroanatomy is not justified. For example, effects within the left IFG are sometimes interpreted as reflecting the engagement of executive resources (e.g., Garbin et al., 2010), and other times as reflecting the engagement of linguistic resources (e.g., Rodríguez-Pujadas et al., 2013). Such flexibility in interpretation is clearly undesirable. Functional localization helps to unambiguously identify the MD vs. the language-selective portions of the left IFG (Fedorenko & Blank, 2020). The same holds for other areas of the association cortex, most of which are highly heterogeneous, containing numerous distinct areas in close proximity to one another.

And third, the use of the same functional localizer paradigms across individuals, studies, and labs enables the establishment of a cumulative research enterprise—the cornerstone of robust and replicable science. This general approach has been de rigueur in other fields, like vision (e.g., Kanwisher et al., 1997) from the earliest days of brain imaging research, and more recently, social cognition (e.g., Saxe & Kanwisher, 2003) and language (Fedorenko et al., 2010). Adopting this approach in the study of executive functions in bilingualism is likely to lead to greater clarity and consensus because of the greater ease of comparing and replicating findings across studies.

Another important feature of our study, which was not present in any prior study, is the use of neural markers that have been previously established (a) to be stable within individuals, (b) to vary across individuals, and (c) to relate to behavioral performance (Assem, Glasser, et al., 2020). This is critically important: A study that does *not* find a difference between bilinguals and monolinguals is impossible to interpret if the relevant neural marker has not been shown to have these properties.

Finally, when arguing for a neural difference between two groups in a particular brain region or network, it is critical to establish the spatial selectivity of the effect. In particular, some effects may be ubiquitously present across the brain and result from nonspecific differences, for example, in the degree of vascularization or arousal. To rule out such effects, we examined a control set of brain areas that respond to the task but are not part of the MD network (primary visual areas). Such control areas have typically been absent from past studies and would be valuable to include in future work.

#### Limitations of scope

Although our study had several methodological advantages over much prior work, it remains a single study probing a particular population of bilinguals: balanced early bilinguals currently residing in the United States. The observation of superior executive abilities in this particular bilingual population is consistent with, but does not directly evaluate, the hypothesis laid out in Blanco-Elorrieta and Pylkkänen (2018). It would help move the field forward if future studies (a) focused on relatively homogeneous groups of bilinguals (e.g., Costa & Santesteban, 2004; Rossi et al., 2017), and/or (b) provided a detailed characterization of their language background and use patterns (de Bruin, 2019).

### Conclusion

In conclusion, we report the first investigation of executive abilities in early bilinguals and matched monolinguals using the kind of robust individual-subject functional localization analytic approach that is likely to yield more interpretable and more easily replicable results than those obtained in past work. We hope that the field of bilingualism research adopts at least some aspects of the approach advocated here, so as to lead to a more robust and cumulative research enterprise.

## DEDICATION

We would like to dedicate this paper to the memory of Albert Costa, who we both knew well and loved as a mentor and a friend. Saima will always be grateful that Albert let her spend her senior year in his lab despite not even being from the same university; his support, mentorship, and guidance helped her not stray away from academia when things got tough. And Ev will forever remember the weekly Friday night partying with Albert and the rest of the “crew” in The Cellar and The People’s Republik during her undergrad years in the Caramazza Lab in the late 1990s and early 2000s.

## ACKNOWLEDGMENTS

We would like to acknowledge the Athinoula A. Martinos Imaging Center at the McGovern Institute for Brain Research at MIT, and its support team (Steve Shannon and Atsushi Takahashi). We thank former and current EvLab members for their help with fMRI data collection (especially Dima Ayyash and Olessia Jouravlev). We also thank Rachel Ryskin and Ted Gibson for helpful discussions. Saima Malik-Moraleda was supported by la Caixa Fellowship LCF/BQ/AA17/11610043. Evelina Fedorenko was supported by the R00 award HD057522, R01 awards DC016607 and DC016950 from NIH, and funds from the Brain and Cognitive Sciences department and the McGovern Institute for Brain Research.

## FUNDING INFORMATION

Saima Malik-Moraleda, “la Caixa” Foundation (https://dx.doi.org/10.13039/100010434), Award ID: LCF/BQ/AA17/11610043. Evelina Fedorenko, National Institutes of Health (https://dx.doi.org/10.13039/100000002), Award ID: HD057522. Evelina Fedorenko, National Institutes of Health (https://dx.doi.org/10.13039/100000002), Award ID: DC016607. Evelina Fedorenko, National Institutes of Health (https://dx.doi.org/10.13039/100000002), Award ID: DC016950.

## AUTHOR CONTRIBUTIONS

**Saima Malik-Moraleda**: Data curation: Lead; Formal analysis: Lead; Investigation: Lead; Methodology: Lead; Validation: Lead; Visualization: Lead; Writing – original draft: Lead; Writing – review & editing: Lead. **Theodor Cucu**: Data curation: Supporting; Formal analysis: Supporting; Investigation: Supporting; Writing – review & editing: Supporting. **Benjamin Lipkin**: Conceptualization: Supporting; Formal analysis: Supporting; Investigation: Supporting; Validation: Equal; Visualization: Supporting; Writing – review & editing: Supporting. **Evelina Fedorenko**: Formal analysis: Equal; Funding acquisition: Lead; Investigation: Supporting; Methodology: Equal; Project administration: Lead; Resources: Lead; Supervision: Lead; Writing – original draft: Equal; Writing – review & editing: Equal.

## TECHNICAL TERMS

Executive processing: A set of cognitive operations required for goal-directed behavior, including working memory, inhibitory control, and selection, among others.
Multiple demand network: A bilateral brain network of frontal and parietal areas that has been implicated in executive processes and linked to fluid intelligence.
Group-constrained subject-specific (GSS) approach: An fMRI approach that enables algorithmic definition of fROIs in individual participants (see Fedorenko et al., 2010).
