# Supplementary material for: The Domain-General Multiple Demand Network Is More Active in Early Balanced Bilinguals Than Monolinguals During Executive Processing
Source: Neurobiol Lang (Camb). 2021 Dec 23;2(4):647–64. doi: 10.1162/nol_a_00058 (PMC10158558; doi:10.1162/nol_a_00058)
Supplement: Supplementary file 1 [file nol-2-4-647-s001.docx]

**Supplementary Materials for Malik-Moraleda et al (2021). The Domain-General Multiple Demand network is More Active in Early Balanced Bilinguals than Monolinguals During Executive Processing.**

**Table of Contents:**

**Table SI-1:** Detailed linguistic profile of participants.

**Table SI-2:** Summary of % BOLD signal change values and standard errors (SE) of the mean by participants in each of the 18 fROIs for each of the contrasts in the experiment.

**Supplementary Information 1: Linguistic Profile of the Participants**

| Group | ID | Age | G | H | N Lang | L1 | | | L2 | | | | |  |
| --- | --- | --- | --- | --- | --- | --- | --- | --- | --- | --- | --- | --- | --- | --- |
|  |  |  |  |  |  | Name | AoA | Prof | Name | AoA | Prof | Parents | School | |
| Biling | 550 | 18 | F | R | 2 | Mandarin | 0 | 5 | English | 0 | 5 | 1 | 1 | |
| Biling | 220 | 27 | F | R | 3 | Russian | 0 | 5 | English | 4 | 5 | 1 | 0 | |
| Biling | 238 | 18 | F | R | 3 | Polish | 0 | 5 | English | 0 | 5 | 1 | 1 | |
| Biling | 339 | 23 | F | R | 3 | Telugu | 1 | 5 | English | 5 | 5 | 1 | 1 | |
| Biling | 360 | 19 | M | R | 2 | Thai | 2 | 4 | English | 2 | 5 | 1 | 1 | |
| Biling | 401 | 19 | M | R | 2 | Spanish | 1 | 5 | English | 1 | 5 | 1 | 1 | |
| Biling | 446 | 35 | M | R | 13 | Polish | 0 | 5 | English | 0 | 5 | 1 | 1 | |
| Biling | 450 | 29 | M | R | 3 | Catalan | 0 | 5 | Spanish | 0 | 5 | 1 | 1 | |
| Biling | 451 | 29 | F | R | 2 | Afrikaans | 0 | 5 | English | 2 | 5 | 1 | 1 | |
| Biling | 459 | 23 | M | R | 4 | Portuguese | 0 | 5 | Spanish | 0 | 4.5 | 1 | 1 | |
| Biling | 467 | 29 | M | R | 3 | Tamil | 1 | 5 | English | 3 | 5 |  |  | |
| Biling | 470 | 31 | M | R | 2 | Hindi | 0 | 5 | English | 4 | 5 | 1 | 1 | |
| Biling | 471 | 29 | M | R | 4 | Hungarian | 0 | 5 | German | 3 | 5 | 1 | 1 | |
| Biling | 478 | 23 | F | R | 2 | Mandarin | 0 | 4 | English | 3 | 5 | 1 | 1 | |
| Biling | 482 | 34 | F | R | 3 | German | 3 | 5 | Romanian | 3 | 5 | 1 | 0 | |
| Biling | 490 | 23 | F | L | 3 | Ukrainian | 0 | 4.5 | Russian | 0 | 5 | 1 | 0 | |
| Biling | 495 | 24 | M | L | 3 | Ukrainian | 0 | 5 | Russian | 0 | 5 | 1 | 1 | |
| Biling | 497 | 35 | M | L | 4 | Spanish | 0 | 5 | English | 3 | 4 | 1 | 1 | |
| Biling | 499 | 17 | M | R | 4 | French | 0 | 5 | English | 0 | 5 | 1 | 1 | |
| Biling | 504 | 26 | F | R | 4 | Hindi | 0 | 5 | English | 2 | 5 | 1 | 1 | |
| Biling | 506 | 30 | F | R | 3 | Hebrew | 0 | 5 | English | 0 | 5 |  |  | |
| Biling | 507 | 30 | F | R | 4 | Danish | 0 | 5 | English | 2 | 5 | 1 | 0 | |
| Biling | 509 | 23 | M | R | 2 | Romanian | 0 | 5 | English | 6 | 5 | 0 | 1 | |
| Biling | 515 | 28 | F | R | 3 | Nepali | 0 | 5 | English | 2 | 5 | 1 | 0 | |
| Biling | 519 | 28 | M | R | 4 | Nepali | 1 | 5 | English | 5 | 5 | 1 | 1 | |
| Biling | 527 | 21 | M | R | 4 | Romanian | 0 | 5 | English | 6 | 4.5 | 1 | 1 | |
| Biling | 534 | 28 | F | R | 4 | Spanish | 0 | 5 | English | 6 | 5 | 1 | 1 | |
| Biling | 546 | 31 | M | R | 3 | Serbocroatian | 0 | 5 | English | 5 | 5 | 0 | 1 | |
| Biling | 561 | 30 | F | R | 4 | Arabic | 0 | 5 | English | 5 | 5 | 0 | 1 | |
| Biling | 565 | 22 | F | R | 5 | Lithuanian | 0 | 5 | English | 4 | 5 | 0 | 0 | |
| Biling | 579 | 25 | M | R | 2 | Lithuanian | 0 | 5 | English | 6 | 5 | 1 | 1 | |
| Biling | 580 | 25 | M | R | 2 | Tagalog | 0 | 5 | English | 0 | 5 | 1 | 0 | |
| Biling | 581 | 24 | M | L | 3 | Nepali | 0 | 5 | English | 0 | 5 | 1 | 1 | |
| Biling | 582 | 25 | F | R | 2 | Russian | 0 | 5 | English | 5 | 5 | 1 | 1 | |
| Biling | 585 | 23 | F | R | 5 | Mandarin | 0 | 5 | English | 6 | 5 | 0 | 1 | |
| Biling | 587 | 25 | M | R | 2 | Mandarin | 0 | 4 | English | 0 | 5 | 1 | 1 | |
| Biling | 591 | 24 | F | R | 3 | Mandarin | 0 | 4 | English | 0 | 5 | 1 | 1 | |
| Biling | 593 | 21 | M | L | 3 | Mandarin | 0 | 5 | English | 0 | 5 | 1 | 1 | |
| Biling | 594 | 25 | M | R | 3 | Mandarin | 0 | 5 | Cantonese | 0 | 5 |  |  | |
| Biling | 595 | 21 | M | L | 2 | Spanish | 1 | 5 | English | 4 | 4.5 | 1 | 0 | |
| Biling | 619 | 40 | M | R | 3 | Finnish | 0 | 5 | Swedish | 0 | 5 | 1 | 1 | |
| Biling | 620 | 29 | M | L | 3 | Irish | 0 | 4.5 | English | 0 | 5 | 1 | 1 | |
| Biling | 628 | 28 | F | R | 3 | Basque | 0 | 5 | Spanish | 0 | 5 | 1 | 1 | |
| Biling | 634 | 28 | F | R | 3 | Latvian | 0 | 5 | English | 0 | 5 |  |  | |
| Biling | 637 | 20 | M | L | 2 | Arabic | 0 | 5 | English | 1 | 5 | 1 | 1 | |
| Biling | 662 | 18 | M | R | 2 | Spanish | 0 | 5 | English | 4 | 5 | 1 | 1 | |
| Biling | 714 | 26 | M | R | 2 | Sinhalese | 0 | 5 | English | 5 | 4 |  |  | |
| Biling | 752 | 20 | M | R | 2 | Mandarin | 0 | 5 | English | 0 | 5 | 1 | 1 | |
| Biling | 799 | 27 | M | R | 3 | Gujarati | 0 | 4.5 | English | 0 | 5 |  |  | |
| Biling | 804 | 19 | F | L | 3 | Swahili | 0 | 5 | English | 0 | 5 |  |  | |
| Biling | 805 | 25 | F | R | 2 | Russian | 0 | 5 | English | 4 | 5 | 0 | 1 | |
| Biling | 809 | 28 | M | R | 5 | Basque | 2 | 5 | Spanish | 0 | 5 | 1 | 0 | |
| Biling | 810 | 28 | F | R | 3 | Marathi | 0 | 5 | English | 2 | 5 | 1 | 1 | |
| Biling | 816 | 19 | F | R | 3 | Hebrew | 0 | 4 | English | 0 | 5 | 1 | 1 | |
| Monoling | 526 | 25 | F | L | 1 | English | 0 | 5 |  |  |  |  |  | |
| Monoling | 563 | 20 | F | R | 1 | English | 0 | 5 | Spanish | 12 | 2 | 0 | 1 | |
| Monoling | 563 | 20 | F | R | 1 | English | 0 | 5 | Spanish | 12 | 2 | 0 | 1 | |
| Monoling | 553 | 19 | M | R | 1 | English | 0 | 5 |  |  |  |  |  | |
| Monoling | 769 | 24 | F | R | 1 | English | 0 | 5 |  |  |  |  |  | |
| Monoling | 214 | 25 | M | R | 1 | English | 0 | 5 | French | 12 | 1.5 |  |  | |
| Monoling | 492 | 23 | M | R | 1 | English | 0 | 5 |  |  |  |  |  | |
| Monoling | 767 | 27 | M | R | 1 | English | 0 | 5 |  |  |  |  |  | |
| Monoling | 261 | 25 | M | R | 1 | English | 0 | 5 |  |  |  |  |  | |
| Monoling | 296 | 23 | M | R | 1 | English | 0 | 5 |  |  |  |  |  | |
| Monoling | 814 | 24 | F | R | 1 | English | 0 | 5 |  |  |  |  |  | |
| Monoling | 369 | 29 | M | R | 1 | English | 0 | 5 |  |  |  |  |  | |
| Monoling | 684 | 30 | F | R | 1 | English | 0 | 5 | French | 30 | 2 | 0 | 1 | |
| Monoling | 682 | 23 | F | R | 1 | English | 0 | 5 | Spanish | 10 | 2 | 0 | 1 | |
| Monoling | 818 | 24 | M | R | 1 | English | 0 | 5 |  |  |  |  |  | |
| Monoling | 786 | 30 | M | R | 1 | English | 0 | 5 | Spanish | 14 | 2 | 0 | 1 | |
| Monoling | 659 | 19 | M | R | 1 | English | 1 | 5 |  |  |  |  |  | |
| Monoling | 338 | 28 | F | R | 1 | English | 0 | 5 |  |  |  |  |  | |
| Monoling | 734 | 26 | F | R | 1 | English | 0 | 5 |  |  |  |  |  | |
| Monoling | 448 | 30 | F | R | 1 | English | 0 | 5 |  |  |  |  |  | |
| Monoling | 366 | 28 | F | L | 1 | English | 0 | 5 |  |  |  |  |  | |
| Monoling | 557 | 23 | M | R | 1 | English | 0 | 5 | Spanish | 14 | 1 | 0 | 1 | |
| Monoling | 760 | 28 | F | R | 1 | English | 0 | 5 | German | 11 | 2 | 0 | 1 | |
| Monoling | 316 | 26 | F | L | 1 | English | 0 | 5 |  |  |  |  |  | |
| Monoling | 574 | 26 | M | L | 1 | English | 0 | 5 | Hindi | 10 | 2 | 0 | 1 | |
| Monoling | 426 | 32 | M | R | 1 | English | 0 | 5 |  |  |  |  |  | |
| Monoling | 331 | 22 | F | R | 1 | English | 0 | 5 | Spanish | 10 | 2 |  |  | |
| Monoling | 524 | 23 | F | R | 1 | English | 0 | 5 | German | 11 | 2 | 0 | 1 | |
| Monoling | 263 | 25 | M | R | 1 | English | 0 | 5 |  |  |  |  |  | |
| Monoling | 642 | 22 | M | R | 1 | English | 0 | 5 | French | 11 | 2 | 0 | 1 | |
| Monoling | 708 | 25 | M | R | 1 | English | 0 | 5 |  |  |  |  |  | |
| Monoling | 431 | 21 | M | R | 1 | English | 0 | 5 |  |  |  |  |  | |
| Monoling | 781 | 25 | M | R | 1 | English | 0 | 5 | Mandarin | 17 | 2 | 0 | 1 | |
| Monoling | 731 | 22 | M | R | 1 | English | 0 | 5 | Spanish | 14 | 2 |  |  | |
| Monoling | 367 | 19 | F | R | 1 | English | 0 | 5 |  |  |  |  |  | |
| Monoling | 318 | 25 | M | L | 1 | English | 0 | 5 |  |  |  |  |  | |
| Monoling | 505 | 28 | F | R | 1 | English | 0 | 5 |  |  |  |  |  | |
| Monoling | 329 | 28 | F | R | 1 | English | 0 | 5 | Italian | 12 | 2 | 0 | 1 | |
| Monoling | 391 | 56 | M | R | 1 | English | 0 | 5 |  |  |  |  |  | |
| Monoling | 600 | 19 | M | R | 1 | English | 0 | 5 | Spanish | 12 | 2 | 0 | 1 | |
| Monoling | 545 | 19 | F | R | 1 | English | 0 | 5 | Mandarin | 14 | 2 |  |  | |
| Monoling | 782 | 22 | M | R | 1 | English | 0 | 5 |  |  |  |  |  | |
| Monoling | 345 | 27 | M | R | 1 | English | 0 | 5 |  |  |  |  |  | |
| Monoling | 658 | 19 | F | L | 1 | English | 1 | 5 |  |  |  |  |  | |
| Monoling | 795 | 25 | F | R | 1 | English | 0 | 5 |  |  |  |  |  | |
| Monoling | 416 | 27 | F | R | 1 | English | 0 | 5 | French | 20 | 2 | 0 | 1 | |
| Monoling | 330 | 27 | M | R | 1 | English | 0 | 5 | Spanish | 12 | 1 | 0 | 1 | |
| Monoling | 824 | 26 | F | R | 1 | English | 0 | 5 |  |  |  |  |  | |
| Monoling | 744 | 19 | F | R | 1 | English | 0 | 5 |  |  |  |  |  | |
| Monoling | 702 | 36 | F | L | 1 | English | 0 | 5 | Spanish | 14 | 2 | 0 | 1 | |
| Monoling | 407 | 20 | M | L | 1 | English | 0 | 5 | Spanish | 13 | 2 | 0 | 1 | |
| Monoling | 559 | 23 | M | R | 1 | English | 0 | 5 |  |  |  |  |  | |
| Monoling | 361 | 26 | M | R | 1 | English | 0 | 5 |  |  |  |  |  | |
| Monoling | 341 | 36 | F | R | 1 | English | 0 | 5 |  |  |  |  |  | |
| Monoling | 233 | 23 | F | R | 1 | English | 0 | 5 |  |  |  |  |  | |
| Monoling | 375 | 27 | M | R | 1 | English | 0 | 5 |  |  |  |  |  | |

Table SI-1: Detailed linguistic profile of participants, including the Group to which they were assigned (Bilig(ual), Monoling(ual)), lab-internal participant ID (for ease of potential cross-referencing with other datasets from our lab), age at the time of testing, gender (G), handedness (H), number of languages spoken (N Lang) (only languages with self-reported proficiency higher than 4 were included here), first language listed by the participant (L1), age of acquisition of the first language (AoA), self-reported proficiency for the first language (on a scale from 1-5) (Prof), second language listed by the participant (L2), age of acquisition of the second language, self-reported proficiency for the second language (on a scale from 1-5), whether at least one parent spoke the language (Parents), and whether the language was studied in school (School).

**Supplementary Information 2: BOLD Percent Signal Change per fROI**

| **ROI** | **Contrast** | **Bilingual** | | **Monolingual** | | **p-value uncorrected** | **p-value Bonferroni-corrected** |
| --- | --- | --- | --- | --- | --- | --- | --- |
|  |  | **BOLD % Signal Change** | **SE** | **BOLD % Signal Change** | **SE** |  |  |
| LH_IFGop | H>Fixation | 2.46 | 0.18 | 2.11 | 0.21 | 0.10 | 1.00 |
| LH_IFGop | E>Fixation | 1.53 | 0.12 | 1.24 | 0.13 | 0.73 | 1.00 |
| LH_IFGop | H>E | 0.93 | 0.09 | 0.87 | 0.12 | 0.09 | 1.00 |
| LH_MFG | H>Fixation | 3.53 | 0.25 | 2.94 | 0.21 | 0.02 | 0.39 |
| LH_MFG | E>Fixation | 2.17 | 0.17 | 1.84 | 0.15 | 0.24 | 1.00 |
| LH_MFG | H>E | 1.36 | 0.12 | 1.10 | 0.14 | 0.14 | 1.00 |
| LH_MFGorb | H>Fixation | 2.75 | 0.19 | 1.83 | 0.17 | 0.00 | 0.00 |
| LH_MFGorb | E>Fixation | 1.43 | 0.12 | 0.80 | 0.10 | 0.13 | 1.00 |
| LH_MFGorb | H>E | 1.31 | 0.12 | 1.03 | 0.13 | 0.00 | 0.01 |
| LH_PreCG | H>Fixation | 3.24 | 0.21 | 2.49 | 0.17 | 0.00 | 0.01 |
| LH_PreCG | E>Fixation | 1.68 | 0.13 | 1.28 | 0.11 | 0.08 | 1.00 |
| LH_PreCG | H>E | 1.56 | 0.13 | 1.21 | 0.13 | 0.05 | 0.85 |
| LH_ParSup | H>Fixation | 2.83 | 0.20 | 1.96 | 0.17 | 0.00 | 0.00 |
| LH_ParSup | E>Fixation | 2.04 | 0.15 | 1.42 | 0.12 | 0.12 | 1.00 |
| LH_ParSup | H>E | 0.79 | 0.08 | 0.54 | 0.08 | 0.00 | 0.00 |
| LH_ParInf | H>Fixation | 3.29 | 0.17 | 2.60 | 0.18 | 0.00 | 0.00 |
| LH_ParInf | E>Fixation | 2.33 | 0.12 | 1.83 | 0.11 | 0.20 | 1.00 |
| LH_ParInf | H>E | 0.96 | 0.08 | 0.77 | 0.10 | 0.00 | 0.02 |
| LH_SMA | H>Fixation | 1.84 | 0.14 | 1.42 | 0.15 | 0.01 | 0.19 |
| LH_SMA | E>Fixation | 0.96 | 0.10 | 0.66 | 0.10 | 0.43 | 1.00 |
| LH_SMA | H>E | 0.88 | 0.09 | 0.76 | 0.09 | 0.05 | 0.83 |
| LH_ACC | H>Fixation | 1.51 | 0.09 | 1.24 | 0.11 | 0.02 | 0.30 |
| LH_ACC | E>Fixation | 0.90 | 0.06 | 0.68 | 0.06 | 0.58 | 1.00 |
| LH_ACC | H>E | 0.61 | 0.05 | 0.56 | 0.07 | 0.02 | 0.31 |
| LH_Insula | H>Fixation | 1.57 | 0.15 | 1.11 | 0.15 | 0.00 | 0.09 |
| LH_Insula | E>Fixation | 0.83 | 0.11 | 0.50 | 0.10 | 0.38 | 1.00 |
| LH_Insula | H>E | 0.74 | 0.08 | 0.61 | 0.09 | 0.03 | 0.57 |
| RH_IFGop | H>Fixation | 1.58 | 0.13 | 1.40 | 0.13 | 0.20 | 1.00 |
| RH_IFGop | E>Fixation | 0.89 | 0.08 | 0.73 | 0.07 | 0.88 | 1.00 |
| RH_IFGop | H>E | 0.69 | 0.07 | 0.68 | 0.09 | 0.17 | 1.00 |
| RH_MFG | H>Fixation | 2.34 | 0.15 | 1.77 | 0.14 | 0.00 | 0.01 |
| RH_MFG | E>Fixation | 1.48 | 0.10 | 1.02 | 0.09 | 0.45 | 1.00 |
| RH_MFG | H>E | 0.85 | 0.08 | 0.75 | 0.08 | 0.00 | 0.01 |
| RH_MFGorb | H>Fixation | 1.70 | 0.12 | 1.36 | 0.11 | 0.01 | 0.16 |
| RH_MFGorb | E>Fixation | 1.07 | 0.08 | 0.78 | 0.08 | 0.64 | 1.00 |
| RH_MFGorb | H>E | 0.63 | 0.06 | 0.57 | 0.07 | 0.01 | 0.25 |
| RH_PreCG | H>Fixation | 3.58 | 0.19 | 2.97 | 0.20 | 0.00 | 0.08 |
| RH_PreCG | E>Fixation | 2.43 | 0.14 | 2.00 | 0.14 | 0.29 | 1.00 |
| RH_PreCG | H>E | 1.15 | 0.08 | 0.96 | 0.10 | 0.01 | 0.27 |
| RH_ParSup | H>Fixation | 0.89 | 0.10 | 0.64 | 0.09 | 0.02 | 0.31 |
| RH_ParSup | E>Fixation | 0.45 | 0.06 | 0.21 | 0.06 | 0.90 | 1.00 |
| RH_ParSup | H>E | 0.44 | 0.06 | 0.43 | 0.06 | 0.01 | 0.19 |
| RH_ParInf | H>Fixation | 4.75 | 0.31 | 4.75 | 0.28 | 0.99 | 1.00 |
| RH_ParInf | E>Fixation | 3.42 | 0.23 | 3.48 | 0.19 | 0.80 | 1.00 |
| RH_ParInf | H>E | 1.33 | 0.11 | 1.27 | 0.13 | 0.79 | 1.00 |
| RH_SMA | H>Fixation | 4.72 | 0.26 | 4.44 | 0.25 | 0.29 | 1.00 |
| RH_SMA | E>Fixation | 3.51 | 0.19 | 3.36 | 0.17 | 0.51 | 1.00 |
| RH_SMA | H>E | 1.22 | 0.10 | 1.08 | 0.11 | 0.50 | 1.00 |
| RH_ACC | H>Fixation | 1.31 | 0.10 | 0.96 | 0.10 | 0.00 | 0.04 |
| RH_ACC | E>Fixation | 0.65 | 0.06 | 0.40 | 0.07 | 0.36 | 1.00 |
| RH_ACC | H>E | 0.65 | 0.06 | 0.56 | 0.07 | 0.01 | 0.23 |
| RH_Insula | H>Fixation | 3.32 | 0.21 | 2.96 | 0.20 | 0.10 | 1.00 |
| RH_Insula | E>Fixation | 2.11 | 0.14 | 1.95 | 0.13 | 0.25 | 1.00 |
| RH_Insula | H>E | 1.21 | 0.10 | 1.01 | 0.10 | 0.36 | 1.00 |

Table SI-2: Summary of % BOLD signal change values and standard errors (SE) of the mean by participants in each of the 18 fROIs for each of the 3 contrasts (Hard>Fixation, Easy>Fixation, Hard>Easy) in the two groups (bilingual, monolingual), and the p-values (both uncorrected, and Bonferroni-corrected for the 18 ROIs) after fitting the following LME model per ROI: EffectSize ~ Group + Condition + Group*Condition + (1|Participant).
